# Online Ratings of Urologists: Comprehensive Analysis

**DOI:** 10.2196/12436

**Published:** 2019-07-02

**Authors:** C William Pike, Jacqueline Zillioux, David Rapp

**Affiliations:** 1 Georgetown University School of Medicine Washington, DC United States; 2 Department of Urology University of Virginia Medical Center Charlottesville, VA United States

**Keywords:** online physician ratings, urology, reputation management

## Abstract

**Background:**

Physician-rating websites are being increasingly used by patients to help guide physician choice. As such, an understanding of these websites and factors that influence ratings is valuable to physicians.

**Objective:**

We sought to perform a comprehensive analysis of online urology ratings information, with a specific focus on the relationship between number of ratings or comments and overall physician rating.

**Methods:**

We analyzed urologist ratings on the Healthgrades website. The data retrieval focused on physician and staff ratings information. Our analysis included descriptive statistics of physician and staff ratings and correlation analysis between physician or staff performance and overall physician rating. Finally, we performed a best-fit analysis to assess for an association between number of physician ratings and overall rating.

**Results:**

From a total of 9921 urology profiles analyzed, there were 99,959 ratings and 23,492 comments. Most ratings were either 5 (“excellent”) (67.53%, 67,505/99,959) or 1 (“poor”) (24.22%, 24,218/99,959). All physician and staff performance ratings demonstrated a positive and statistically significant correlation with overall physician rating (*P*<.001 for all analyses). Best-fit analysis demonstrated a negative relationship between number of ratings or comments and overall rating until physicians achieved 21 ratings or 6 comments. Thereafter, a positive relationship was seen.

**Conclusions:**

In our study, a dichotomous rating distribution was seen with more than 90% of ratings being either excellent or poor. A negative relationship between number of ratings or comments and overall rating was initially seen, after which a positive relationship was demonstrated. Combined, these data suggest that physicians can benefit from understanding online ratings and that proactive steps to encourage patient rating submissions may help optimize overall rating.

## Introduction

Recent data demonstrate that most Americans use the internet to search for health information [1-3]. In addition, a large percentage of patients obtain information about physicians through internet resources and identify online websites as important in their choice of health care providers [4,5]. A prior study evaluating patient trends reported that 59% of the US population reported physician-rating websites (PRWs) to be somewhat important in choosing their health care providers [4]. At the same time, there has been a tremendous growth in the number of PRWs [6]. There exist at least 28 PRWs that display information about physician training and allow users to rate physician or staff characteristics.

Although criticisms regarding the validity of PRWs are often raised by physicians, these data show the importance of online physician reputations. In addition to the importance of PRWs in guiding patient selection as consumers, online rating systems are also part of a more widespread focus on the patient experience. Accordingly, the Hospital Quality Alliance was established in an effort to promote transparency of care quality reporting [7]. The initiatives of the Hospital Quality Alliance and Medicare are seen in publicly available data focused on core care measures that include patient surveys about their care (Hospital Consumer Assessment of Healthcare Providers and Systems). As such measures of patient experience become more commonly used to assess care quality and influence reimbursement models (eg, value-based purchasing), it becomes even more important that physicians maintain a working knowledge of online patient reviews.

Even so, investigation suggests that many physicians have little familiarity with PRW, do not commonly check their own reviews, and spend minimal time managing their digital reputation [8]. Although reputation management is a frequent focus in commerce and marketing literature, little is written about online reputation management of physicians. Suggestions for optimization of online ratings within the general literature include actively encouraging patients to submit ratings and responding to negative comments online [9].

Within the urology literature, we could identify only two studies focused on the assessment of online ratings [10,11]. Thus, we sought to comprehensively assess online ratings in a large cohort of urologists. Specific study aims included the assessment of the relationship between number of ratings and the overall mean rating. We hypothesized that number of ratings would demonstrate a positive correlation with overall ratings as this may reflect initiatives by certain physicians to actively manage their reputation and encourage patients to submit online comments or ratings. We also sought to assess the distribution of ratings and assess for a correlation between individual physician and staff characteristic ratings and overall rating.

## Methods

We conducted an analysis of urologic physician ratings and related information on the website Healthgrades. Data retrieval was facilitated using Java (version 8). Specific focus was placed on aggregating data related to physician and staff ratings, including number and distribution of ratings, number of comments, physician performance characteristics, as well as office and staff performance characteristics.

In brief, overall physician ratings are provided as a score between 1 and 5 (1=poor; 5=excellent). Ratings are also available for a specific physician (trustworthiness, explains conditions well, answers questions, time well spent) and staff (scheduling, office environment, staff friendliness) performance variables. Each of these physician and staff performance variables is also rated on a score of 1 to 5.

Inclusion and exclusion criteria were designed in an effort to focus on a cohort of actively practicing urologists and exclude those that may be in residency, retired, or deceased. Accordingly, physicians with a known age of 35 to 74 years were included. These age criteria were selected after an initial data review of age-related ratings distribution, which revealed that the majority of physicians with ages younger than 35 or older than 74 years had zero ratings. Physicians without data specifically detailing age or an age estimation (years out from medical school) were also excluded.

Analysis first focused on descriptive statistics to assess overall physician rating, number of ratings or comments per physician, and ratings related to specific physician and staff performance variables. Variables are presented as mean and standard deviation. We then assessed for a Pearson correlation between physician and staff performance variables and overall physician rating. Finally, we performed a best-fit analysis to assess for an association between number of physician ratings or comments and overall rating. Statistical analysis was performed using R (version 3.4.1). All tests were performed with α=0.05. The University of Virginia (Charlottesville, VA) institutional review board determined that this study met the criteria for nonhuman research (IRB #: 20592).

## Results

Data were retrieved for 14,430 urologists, of which 9921 met the inclusion criteria and were included in study analysis. A total of 99,959 ratings and 23,492 comments were seen across 9921 urologists. The mean number of ratings and comments per urologist was 10.1 (SD 4.3) and 2.4 (SD 6.0), respectively. In addition, a significant range in number of ratings (0-395) and comments (0-241) per urologist was seen. Analysis demonstrated that 1554 of 9921 (15.66%) and 4077 of 9921 (41.09%) of physicians had zero ratings and zero comments, respectively.

**Figure 1:**
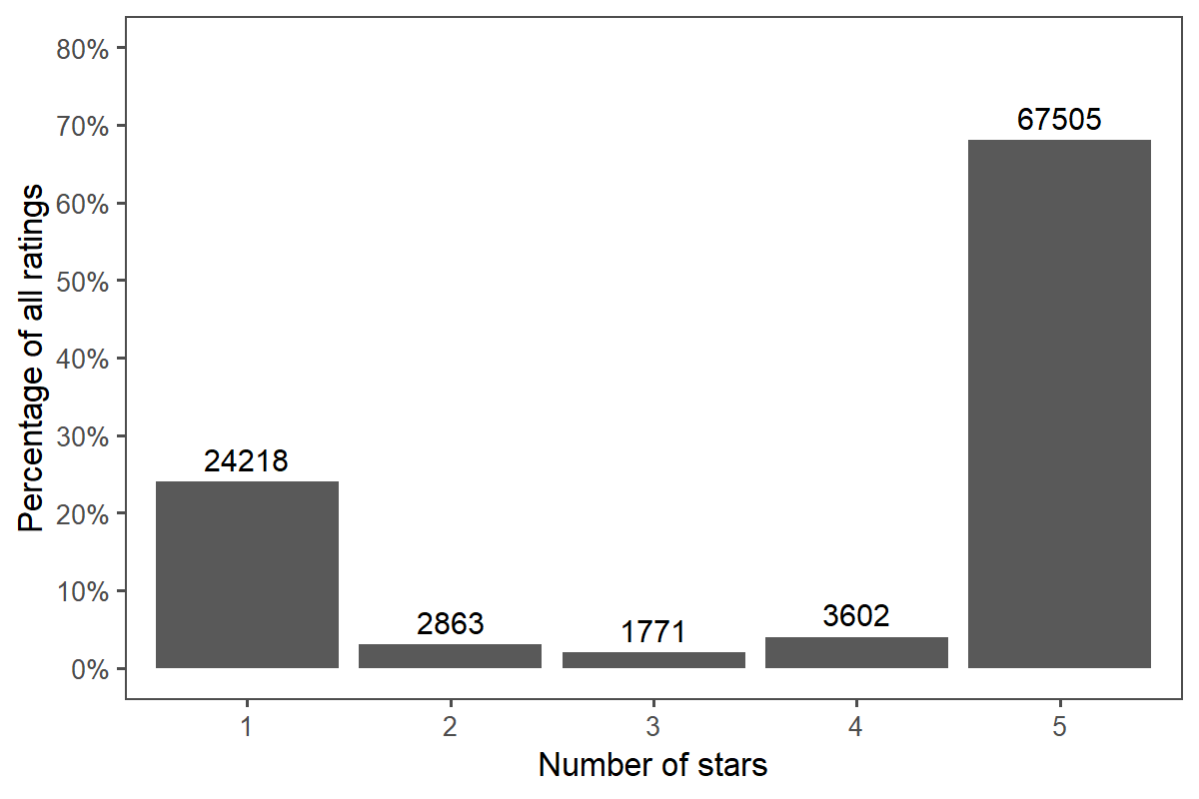
Distribution of ratings of urologists on a physician-rating website (N=99,959).

The distribution of ratings is showed in Figure 1. The vast majority of ratings were either 5 (“excellent”) (67.53%, 67,505/99,959) or 1 (“poor”) (24.22%, 24,218/99,959). Mean overall physician rating was 3.9 (SD 1.7). Physician and staff performance variable statistics and their correlation with overall ratings are detailed in Table 1. All physician and staff performance ratings demonstrated a positive and statistically significant correlation with overall physician rating. The statistical coefficients (*R* value) for trustworthiness and answers questions were highest. Physician measures had higher correlations with overall rating than did staff measures.

**Figure 2:**
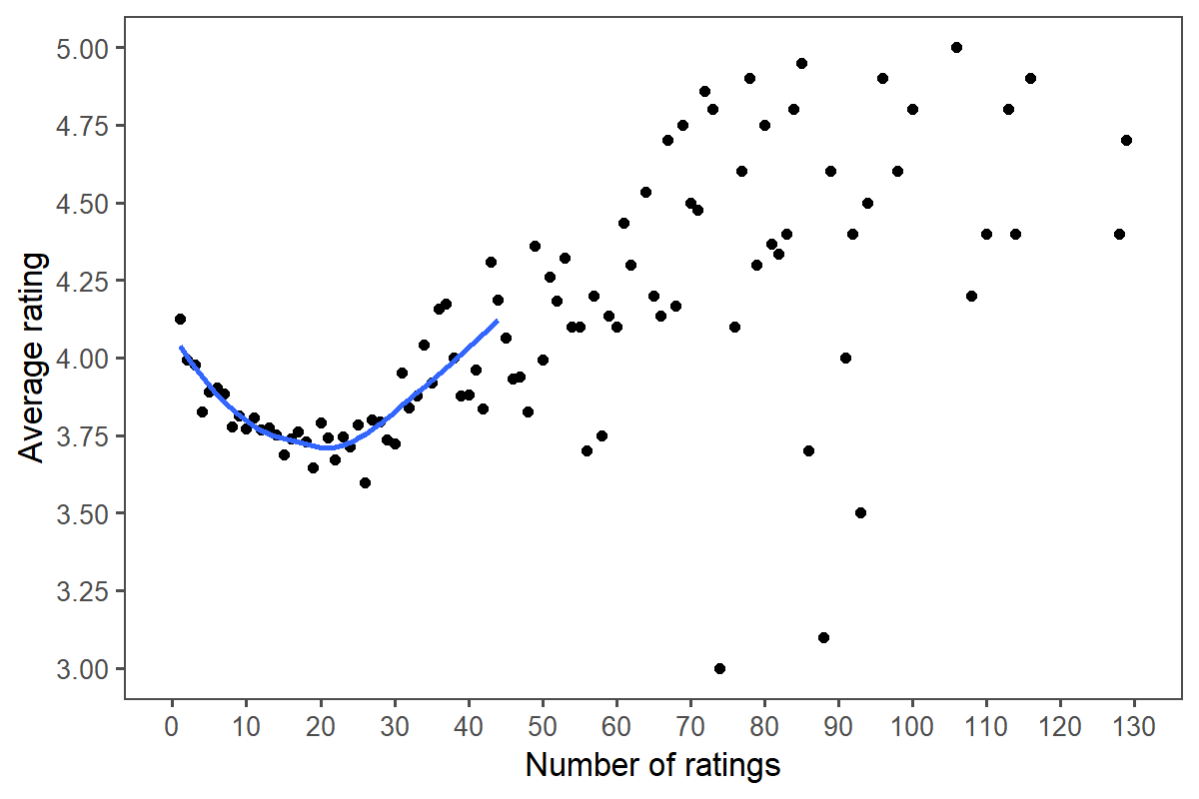
Relationship between number of ratings and overall physician rating.

**Figure 3:**
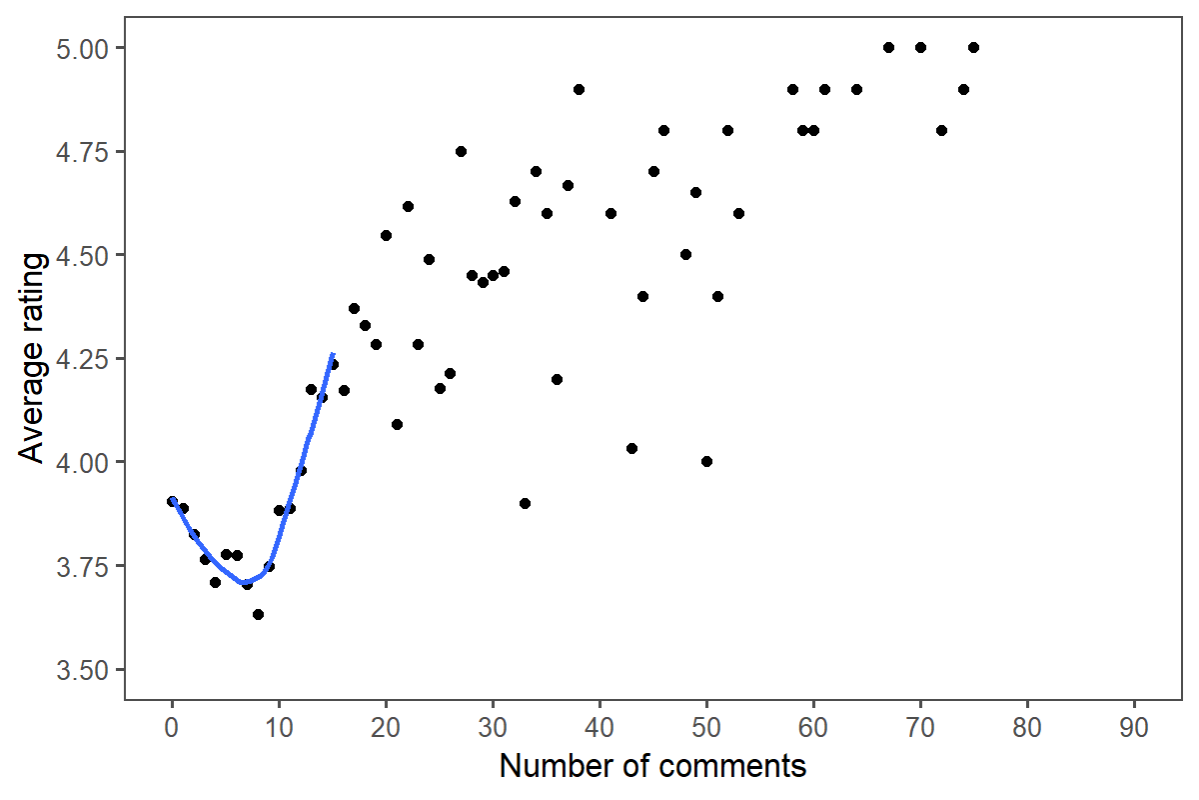
Relationship between number of comments and overall physician rating.

Best-fit analyses of the relationship between number of ratings and overall physician rating as well as between number of comments and overall physician rating are shown in Figures 2 and 3 with locally weighted smoothing added for clarity. A negative relationship between the number of ratings and overall rating was seen until physicians achieved 21 ratings; thereafter, a positive relationship was seen. Similarly, a U-shaped relationship was seen when assessing the relationship between number of comments and overall rating, with the transition point being six comments.

**Table 1:** Physician and staff performance variables and correlation with overall ratings.

Performance variable		Rating, mean (SD)	Correlation with overall rating^a^, *R*	*P* value
**Physician measures**				
	Trustworthiness	3.96 (0.89)	.965	<.001
	Explains conditions well	3.99 (0.88)	.953	<.001
	Answers questions	4.20 (0.70)	.957	<.001
	Time well spent	4.02 (0.76)	.947	<.001
**Staff measures**				
	Scheduling	4.09 (0.75)	.805	<.001
	Office environment	3.96 (0.88)	.796	<.001
	Staff friendliness	3.98 (0.88)	.817	<.001

^a^Overall rating, mean (SD)=3.87 (1.72).

## Discussion

This study reveals several important findings. To our knowledge, this is the largest study comprehensively analyzing online ratings of a nationwide sample of urologists. Overall, more than 80% of urologists had at least one rating, demonstrating the use of PRWs by patients. These data are consistent with a prior study evaluating urologist ratings, which highlighted only that a small percentage of physicians do not have ratings associated with their profiles [10,11]. Interestingly, other investigations demonstrated that a large percentage of physicians overall have no ratings, but that specialists are twice as likely to have online ratings when compared with generalists [6,12]. Combined with our investigation, these data highlight the high utilization of PRWs within the surgical community and the need to demonstrate awareness with one’s online ratings.

Second, the vast majority of ratings submitted were either excellent (5) or poor (1), with almost one-quarter of ratings being 1. Other studies have described ratings distributions, with most being positive. Kadry and colleagues [13] demonstrated that approximately two of three patient reviews were favorable across 23 specialties. Lagu et al [14] found that 88% of ratings were positive for both generalists and specialists (as defined by a rating of 3 or greater on a 4-point scale). A prior study also demonstrated that most comments on PRWs are positive [15]. Generally, our findings are consistent with these prior investigations suggesting that most ratings are either excellent or poor [16]. Notably, in review of physician ratings on RateMD.com, Gao et al [17] found that 42% of the ratings were between 2 and 4 on a 5-point scale. This differs significantly from our results, which demonstrate an extreme dichotomy of ratings.

Further, all performance variables assessed strongly correlated with overall physician rating. This finding suggests that these variables all influence a patient’s overall satisfaction with the visit. A prior study demonstrated a statistically significant correlation between staff and physician ratings [17]. In addition, Kadry and colleagues [13] reported a strong correlation between a diverse number of dimensions of the patient appointment and overall rating. In this study, dimensions assessed included communication skills (eg, listens and answers questions) and access (eg, ease of appointment, punctuality). Our analysis adds to this literature because it assesses further variables that may influence patient satisfaction.

Most notably, our analysis demonstrated a U-shaped relationship between number of ratings and overall mean rating. A similar relationship was observed in the relationship between number of comments and overall mean rating. Accordingly, before achieving 21 patient ratings, a negative relationship was demonstrated between number of ratings and overall mean rating, followed by a positive relationship. Similarly, a rating nadir was seen at six comments, after which a positive relationship was seen. We hypothesize that this relationship is created from the significant impact that a single poor rating can have on the overall mean rating when there are few ratings. Prior opinion supports this theory, suggesting that in cases in which there are few ratings, one outlying value or comment can have a disproportionately large influence [18].

Combined, these findings emphasize the importance of active knowledge and management of online reputation by urologists. Experience related to online reputation management suggests that a single negative review likely has a greater influence than multiple positive evaluations [19]. Despite this fact, a large percentage of physicians do not check their online profile [8,20]. Physician criticism of PRWs is understandable given previous studies demonstrating inconsistencies between patient ratings and quality of care [21-25]. However, given data demonstrating the rapid increase in the utilization of PRWs by patients, it is important that physicians have a working knowledge of their online reputation. Further, patients are increasingly providing online reviews of hospitals and treatment centers [26]. As patient satisfaction with physicians can also be influenced by the patient experience at the hospitals where they offer care, physician awareness of these facility reviews is also important.

In addition, active steps by physicians should be considered to help optimize ratings. Foremost, our data suggest that efforts should focus on building total volume of reviews on PRWs. Suggested approaches involve the use of collateral to solicit reviews, including patient cards, videos, and emails [9]. In addition, patients can be encouraged to complete online ratings and surveys at the time of encounter, thus offering a more proactive approach [8]. Finally, appropriately addressing negative comments or providing personalized review responses is suggested as a potential method of demonstrating physician focus on the patient experience to other potential patients visiting the PRW [27]. This is important given a study showing that only 39% of physicians agree with their profile ratings [20]. Further study is ongoing at our institution to assess specific methods of optimizing patient engagement and ratings.

Beyond commercial PRWs, focus on additional online forums can help optimize physicians’ digital reputations. One such method includes using online professional networking websites (eg, Doximity) to publish professional accomplishments [28]. The creation of a personal online blog by physicians offers another technique to share information with patients [8]. Further, the use of social media pages (eg, Facebook) can be an effective method of managing online reputation. Indeed, social media presence, such as Facebook followers, has been shown to correlate with US News and World Report reputation score [29]. Finally, utilizing noncommercial PRWs can also be valuable because the percentage of positive comments has been shown to be higher on health systems’ online review websites when compared to commercial PRWs [15].

Study limitations include the study focus on ratings from a single PRW. Accordingly, the findings in this study may not be representative of trends across all PRWs. Healthgrades was selected because it is the most widely used PRW [13]. Supporting this trend is a prior systematic review showing that Healthgrades is the most widely selected PRW assessed in published investigations [26]. In addition, our study aim was to systematically assess ratings information across a large cohort of urologists through use of Java programming. Indeed, our cohort consisted of almost 10,000 urology profiles. However, given this methodology, analysis of text comments was not possible. Similarly, given the variability in rating scales and domains across the PRW, the inclusion of multiple PRWs and systematic comparison is difficult. Novel methods of automated analysis of text reviews have been recently reported and may allow for a more comprehensive study of text-based patient reviews in the future [30,31].

Nonetheless, we believe our study conclusions are strengthened by the large size of our cohort. Prior systematic review of studies on patient online reviews demonstrated that, in general, the number of providers with online reviews reported in investigations represented only a small percentage of the total workforce [26]. Recent data from the American Board of Medical Specialties reports 13,039 board-certified urologists [32]. Although there may be additional urologists without board certification, these data suggest that our study captured over 75% of all urologists within the United States and highlight the significance of our cohort size. In addition, our data represent a large and diverse sample size across various regions, practice types, physician ages, and other physician characteristics. Future study is ongoing to assess the potential relationship between these variables and online ratings. Such study is important given conflicting data regarding the relationship between physician characteristics (such as gender, practice experience, and academic productivity) and patient online ratings [26].

In conclusion, our study demonstrates that most online urologist profiles have received ratings. Further, a dichotomous rating distribution is seen, with more than 90% of ratings being either poor or excellent. A negative relationship between number of ratings and overall rating is initially seen, following which a positive relationship is demonstrated. Combined, these data suggest that physicians can benefit from understanding ratings associated with their online profile and that proactive steps to optimize their rating may be helpful.

## Abbreviations

PRW: physician-rating website
